# Experimental Study on the Status of Maize Mycotoxin Production in Farmers’ Grain Storage Silos in Northeastern China

**DOI:** 10.3390/toxins13110741

**Published:** 2021-10-20

**Authors:** Jinsong Zhang, Yan Xu, Taogang Hu, Changpo Sun, Wenfu Wu

**Affiliations:** 1 Department of Agricultural Engineering, College of Biological and Agricultural Engineering, Jilin University, Changchun 130022, China; jluzjs@jlu.edu.cn (J.Z.); xuyan@jlu.edu.cn (Y.X.); 2 Department of Grain Science and Technology, Jilin Business and Technology College, Changchun 130507, China; lkyhtg@163.com; 3 Standards and Quality Center of National Food and Strategic Reserves Administration, No.11 Baiwanzhuang Avenue, Xicheng District, Beijing 100037, China

**Keywords:** farmers’ grain storage silos, absolute water potential, ventilation and drying, mycotoxins, contamination distribution

## Abstract

The scientific rationality of farmers’ grain storage technology and equipment is crucial for the biosecurity of grain in the main grain-producing areas represented by Northeast China. In this paper, four farmer grain storage mock silos of different widths were used as a means to track an experimental cycle of grain storage. The absolute water potential of corn in all four silos at the beginning of the experiment was greater than the absolute water potential of air, prompting moisture migration from the grain interior to the air and down to about 14%. Moisture was influenced by wind direction, and moisture decreased faster with better ventilation on both sides of the grain silos. Therefore, grain silo width has a significant effect on the drying effect under naturally ventilated conditions of maize ears. This research focused on the determination and assessment of mycotoxin contamination under farmers’ storage grain conditions and analyzed the effect of silo structure on the distribution of mycotoxin contamination. When the width was too large, areas of high mycotoxin infection existed in the middle of the grain silo, and ventilation and tipping could be used to reduce the risk of toxin production. This study proved that reasonable farmer grain storage techniques and devices in Northeast China can effectively protect grain from mycotoxin contamination.

## 1. Introduction

Maize is one of the most widely grown crops in the world and is cultivated in more than 170 countries and regions worldwide. As the world’s most productive food crop, it can be used in a wide variety of food and industrial products and is also the predominant forage grain [1]. From 1967 to 2019, world maize production increased from 272 million tons to 1.11 billion tons. With the increase in world population, the demand for maize in developing countries will also increase significantly. As a basic staple food, maize of excellent quality needs to maintain high standards in terms of organoleptic, nutritional, and microbiological quality. However, nutrient and dry matter losses are usually caused by spoilage molds, and mycotoxin contamination may occur at the pre-harvest and post-harvest stages [2,3,4]. Important fungal toxins associated with maize include aflatoxins, which are produced by *Aspergillus flavus* (AFs); deoxynivalenol (DON), which belongs to the monoterpene group of toxins and is mainly produced by *Fusarium graminearum* and *Fusarium pink*, also known as vomitoxins due to their characteristic ability to induce vomiting in animals; fumonisins (FMs), produced by *Fusarium verticillioides* and *Fusarium proliferatum*; and zearalenone (ZEA), produced by *Fusarium graminearum* [5,6,7]. Climate and storage conditions have a significant impact on the production of mycotoxins, and if maize is grown in the tropics and subtropics with high temperatures and humidity, ear and grain rots caused by a variety of fungi are prevalent, causing farmers to suffer substantial economic losses [8]. If storage conditions are not well managed, the large increase in the number of insects and microorganisms in maize makes it more susceptible to fungal attack, thus greatly increasing the chance and extent of contamination by mycotoxins [2].

In response to the paradox of current and future food shortages, governments often seek to increase food availability by increasing food production. However, another important measure is to reduce food losses to balance the growing demand for food production, which often does not receive the attention it deserves [9,10,11,12]. In East and Southern Africa, for example, maize, the most important staple food, has experienced severe post-harvest losses in the past, resulting in reduced income for farmers [13]. Maize is infested with molds during storage, producing large amounts of fungal toxins, thereby losing its edible value [14]. Farmers still use many traditional storage methods to preserve grains, such as ground storage (ground-level grain), bags, baskets, or jars. However, these measures often do not guarantee the grain’s protection from insects, pests, rodents, and molds [15].

Chinese farmers’ grain storage accounts for about 50% of the total national grain production, about 250 million tons. The average grain storage per household is about 1200 kg, but there are large regional differences. In Jilin and Heilongjiang Provinces, which are the main grain-producing areas in the northeast, the average grain storage of farmers is more than 5000 kg, and in Liaoning Province, the average household storage is 3000–5000 kg. Due to the lack of suitable grain storage equipment and storage management techniques in most areas of the country, the level of storage is low, and a large amount of grain is lost due to factors such as rodents, insects, and mildew. According to a sample survey conducted by the National Grain Bureau of China, the average loss rate of grain stored by farmers nationwide is about 8%, with an annual loss of about 40 billion kg of grain. Among the main varieties of grain stored by farmers, corn has the highest loss rate, with an average of about 11%; rice is about 6.5% and wheat is about 4.7%. Therefore, safe storage is crucial, and it directly affects the overall quality and safety of grain as well as the income of the majority of farmers. The main causes of losses are mold (contributing about 30%) and insect damage (about 21%) [16]. Farmers’ grain storage losses in the northeast are also more serious, with an average of about 10.2%. The poor storage conditions have caused a serious deterioration in grain quality, which poses a great potential hazard to food and food safety in China [17].

To facilitate grain storage, different grain storage devices have been designed for farmers around the world. The metal silo is a cylindrical structure made of galvanized iron. It has been shown to be effective in protecting harvested grain not only from storage insects but also from pests such as rodents, insects, and birds [18,19]. Although metal silos suffer from poor airtightness and high costs, they have become one of the key technologies for effective post-harvest management of grains, thereby improving food security for smallholder farmers [20]. In recent years, closed grain storage units have been increasingly promoted in Asia and Africa [21,22]. This device prevents moisture loss from the grain and limits gas exchange, thus changing the atmosphere inside the device. Purdue Improved Crop Storage (PICS) storage bags have been developed and promoted as a way to address the grain storage problems faced by farmers in developing countries. This is a sealing technology that works by strictly limiting the inflow of oxygen into the bulk grain. PICS bags can reduce the growth of insect populations in storage by 98% and can reduce grain losses due to insects and molds in storage to less than 1% while maintaining their quality for months or longer [23,24,25]. In addition to this, there are plastic silos [24], grain safety bags [26], and Grain Pro Super bags [27], which are containers with a multilayer composite technology for better gas-tight storage.

The most used grain storage devices in Northeast China are assembled corn ear storage silos. To meet the characteristics of rapid ventilation and water reduction, grain storage silos are designed as a single-side ventilated steel skeleton metal mesh structure. Such silos are conducive to natural ventilation and precipitation, and solve the problems of high moisture and easy mildew of newly harvested maize ears [28,29]. To save the land and increase corn storage capacity, corn ear storage silos can be designed with a hollow central silo structure by combining the “chimney effect” of air convection heat transfer with traditional natural ventilation [28]. The moisture content of corn at harvest is generally 25–33% and must be reduced to safe moisture before storage. The use of hot air drying can reduce the moisture in a short period, but corn treated with hot air has flaky kernels and reduced quality. Maize drying technology on silos has been greatly developed. Natural low-temperature grain storage can be achieved by taking natural ventilation and freezing precipitation in autumn and winter [30]. This can inhibit the respiration intensity of grain, delay aging, and reduce the loss of stored grain. Reasonable control of the in-silo and out-silo periods will affect the final quality of corn and the safety of grain storage.

Currently, there are few studies on the effects of farmer storage silos on maize mycotoxin production in Northeast China [31,32]. Therefore, this study conducted a natural ventilation test on corn ears to analyze water diffusion and temperature distribution of corn ears during natural precipitation and the effects of these factors on the production of mycotoxins. Based on the mining of biosecurity data from previous farmers’ grain storage, it is shown that farmers’ scientific grain storage can still adapt to the biosecurity requirements of grain in the new era and is an effective means to ensure the supply of high-quality grain materials to the market.

## 2. Results

### 2.1. Moisture Changes in Grain Piles

Table 1 shows the average moisture values of corn ears in each silo during the storage period. From the data in the table, it could be seen that the average moisture in silos 2, 3, and 4 had dropped to the safe moisture level specified in the national standard. Silo 1, which was wider and relatively poorly ventilated, also had its average moisture content reduced to 15.06% at the end of the test. Therefore, the use of rectangular silos with a reasonable structure for the storage of corn ears could reduce their moisture content to a safe moisture level after about 4 months of natural ventilation.

### 2.2. Variation and Distribution of Absolute Water Potential in Grain Piles

The concept of water potential was introduced into the field of grain drying in 2003 and applied to construct a grain drying model. It is mainly due to the fact that water migration is related to the existence of water potential difference between the inside and outside of grain particles. In 2007, Wenfu Wu numerically solved a water potential-based model for drying maize using a difference algorithm. The results of the solution were compared with experimental data and showed that the model could be applied to the simulation of the vacuum drying process of maize [33]. Later, the Laplace transform was further applied to derive an exact algebraic model from the dichotomous model of drying based on water potential [34], and a theoretical model of water potential applied to the moisture change process in naturally ventilated corn cob storage silos was established. In 2012, Zidan Wu et al. proposed a method to manage and control mechanical ventilation operations in grain silos using water potential maps. The concept and principle of water potential were further introduced into the management of the mechanical ventilation of grain silos by drawing an absolute water potential map of grain [35]. In 2016, Zhe Liu conducted a simulation and experimental study of the deep bed drying process of grain based on the conceptual model of water potential [36]. After the development of the above research work, the absolute water potential of air in the grain pile, *E_j__a_* (Equation (1)), and the absolute water potential of grain, *E_jg_* (Equation (2)), were defined. For the correlation coefficients in the equations (i.e., *A*_1_, *A*_2_, *B*_1_, *B*_2_, and *D*_0_), data from the equilibrium moisture isotherm model of maize were used [37,38]. These parameters were obtained by fitting desorption and adsorption equations by nonlinear regression analysis using self-determined equilibrium moisture/equilibrium relative humidity data of maize by the Academy of Sciences of China Grain Bureau. This provides a theoretical basis for grain drying, grain depot ventilation decision, and conditioning storage. The absolute water potential lines for air and grain can be plotted in the water potential diagram. The absolute water potentials of air and grain were used to calculate the absolute water potential of corn during natural ventilation and the absolute water potential of air under experimental conditions, respectively, to assess the absolute water potential variation and distribution of grain piles and the drying effect of grain silos.

The evaporation of liquid water vaporization inside the grain needs to gain energy and overcome the resistance of the grain for the moisture to migrate from the grain interior to reach the grain surface. Additionally, the evaporative migration of water from the surface of the grain to the air must overcome the binding energy between the water and the grain. In this study, based on the previous theoretical basis, the absolute water potential was used to characterize the water migration due to the energy exchange caused by any kind of unbalanced potential between the inside of the grain and the external environment, such as temperature, pressure, and moisture content. If the absolute water potential of the corn is greater than the absolute water potential of the ambient air, the grain moisture migrates to the air and the grain is in a state of desorption. If the absolute water potential of the grain is less than the absolute water potential of the air, the moisture in the air migrates into the grain kernels to produce adsorption.

**Figure 1:**
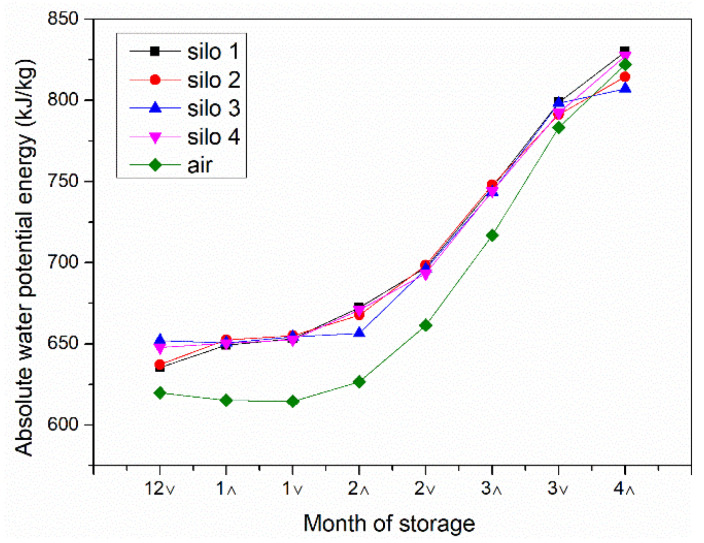
Comparison of water potential of corn in the silos with air water potential. The horizontal numbers represent months, “∧” represents the first half of the month, and “∨” represents the second half of the month.

The absolute water potential values of air and corn during the test were obtained using Equations (1) and (2), as shown in Table 2 and Table 3. The trend of the absolute water potential of the corn and the external environment in each silo during the whole storage process is shown in Figure 1. At the beginning of the experiment, the absolute water potential of corn in all four silos was greater than the absolute water potential of air. Under the action of the water potential, the moisture migrated from the inside of the grain to the air, and the moisture content of the corn in the silos gradually decreased, with the average moisture content dropping from about 25% at the beginning to about 14%. As the ambient temperature rose, the absolute water potential of both air and corn gradually increased, but the difference between the absolute water potential of corn and the absolute water potential of air gradually decreased. When the absolute water potential of the corn was equal to the absolute water potential of the air, the water molecules did not have enough energy to diffuse from the surface to the surrounding air, and although the ambient temperature was higher the corn moisture was no longer falling.

### 2.3. Detection and Distribution of Mycotoxins in Grain Piles

In each of the four storage silos, 16 toxin sampling points were selected for each silo. Table 4 shows the results of aflatoxin B1 and B2 measurements on the collected samples (Before and after storage, aflatoxins G1 and G2 were not detected, so they were not shown in the table. Aflatoxin B1 and B2 were both non-detected before storage). Table 5 shows the results of the determination of deoxynivalenol (DON) content in the collected samples (413.24 ± 3.57 μg/kg of DON before storage). Table 6 shows the results of the determination of the zearalenone (ZEN) content of the collected samples (25.32 ± 1.36 μg/kg of ZEN before storage).

#### 2.3.1. Aflatoxin Contents and Distribution

**Figure 2:**
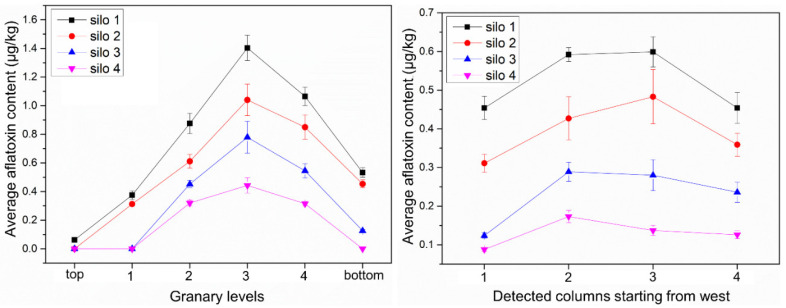
Aflatoxin contents of samples from different locations of grain storage silos.

From Table 4, it could be calculated that the total aflatoxin content of each testing point in each storage silo was between 0 and 1.70 μg/kg. The safe limit range of total aflatoxin content in food in China is <5–10 μg/kg, so the aflatoxin is within the safe level, and the total aflatoxin contamination level can be effectively controlled in farmers’ grain storage silos. Table 7 reflects the average aflatoxin content of each layer in each storage silo. The data for each layer were summed and averaged to obtain the average aflatoxin content. From Table 8, it could be seen that silo 4 among the four storage silos had the best effect in controlling the contamination level of total aflatoxin content. Combined with Figure 2, it also could be seen that the middle layer (i.e., the third layer) and the middle column samples of all silos had higher aflatoxin contents. There were significant differences in aflatoxin levels between the layers of the silos, with a difference of 75.14% between the minimum and maximum values (Table 9). The average aflatoxin level in the third layer of silo 1 reached a maximum of 1.403 μg/kg; therefore, the middle area was more likely to contribute to aflatoxin production during storage.

#### 2.3.2. Deoxynivalenol (DON) Contents and Distribution

**Figure 3:**
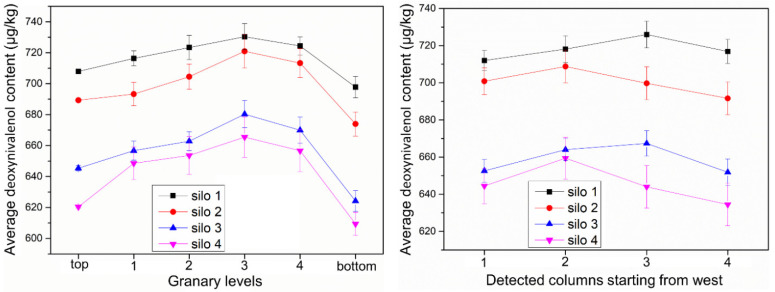
Deoxynivalenol contents of samples from different locations of grain storage silos.

As can be seen from Table 5, the DON content of each testing point of each grain storage silo was between 600 and 750 μg/kg. The safe limit range of DON in food is <1000 μg/kg in China, so the DON content of each silo is within the safe level, and the farmers’ grain storage silo can effectively control DON contamination. Table 10 reflects the average DON content of each layer in each storage silo. The results obtained for each layer in Table 10 were summed and averaged to obtain the average DON contents in Table 11. The data showed that silo 4 had the best effect in controlling the DON contamination level. As can be seen in Figure 3, the DON content of the middle (third) sample was higher. Although silo 1 had the highest level of DON content, there was no significant difference in DON contents between the silos, and the difference between the minimum and maximum values was only 10.05% of the ratio (Table 12). This indicated that DON was mainly produced before entering the silos (DON content was 413.24 ± 3.57 μg/kg before storage), and it was advisable to use measures such as timely harvesting and drying into the silos to mitigate mycotoxin production.

#### 2.3.3. Zearalenone (ZEN) Contents and Distribution

**Figure 4:**
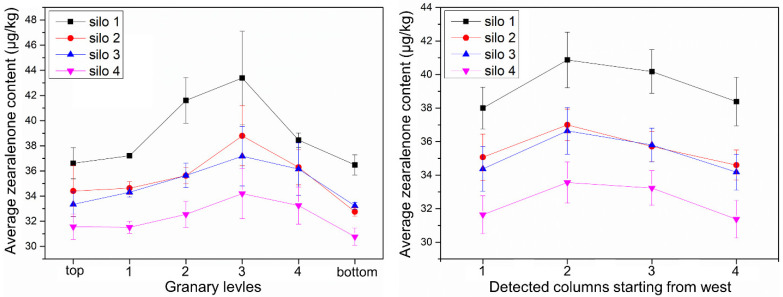
Zearalenone contents of samples from different locations of grain storage silos.

From Table 6, it could be seen that the ZEN content of each testing point was between 30 and 47 μg/kg. The safe limit range of ZEN in food is <60 μg/kg in China, so the ZEN content of each silo is within the safe level. Table 13 reflects the average content of ZEN in each layer in each storage silo. The data for each layer were summed and averaged to obtain the average level of ZEN content, as shown in Table 14, which showed that silo 4 has the best effect in controlling the ZEN contamination level. As can be seen in Figure 4, the ZEN content of the sample from the middle layer (third layer) was higher. There is a significant difference between the ZEN content levels in each layer of the silo, with a minimum and maximum difference ratio of 17% (Table 15).

### 2.4. Mold Rate

The Chinese national standard GB1353–2009 makes clear provisions for mold rate, and raw mold grains need to be ≤2%. Table 16 shows the mold rate of corn in the silos at the end of the experiment. The data indicated that the mold rate in silos 1 and 2 was relatively high and not uniform, while both indicators were better in silos 3 and 4. It could be seen that the structure of the silo had a greater impact on the mold rate. The wider the width of the silo, the more serious the mold, and the fluctuation range of the mold rate was large. Therefore, the structure of the grain storage silo should be reasonably designed to reduce the occurrence of mold. The level of corn mycotoxin content was mainly related to whether the corn was infected with toxin-producing molds. Although there was no significant positive correlation between the number of moldy grains and mycotoxin content, the mold rate could be used to evaluate whether the storage silo design was reasonable from the perspective of whether maize was susceptible to mold infection [39,40].

## 3. Discussion

The vaporization and evaporation of liquid water inside the grain require the acquisition of energy to overcome the internal resistance of the grain. Only then can the moisture migrate from inside the grain to reach the surface of the grain. Additionally, the evaporative migration of moisture from the grain surface to the air must overcome the binding energy between the moisture and the grain. The presence of an imbalance between the inside of the maize kernel and the external environment in terms of temperature, pressure, and moisture can cause energy exchange and result in water migration. In this study, absolute water potential was used to characterize this energy exchange. The activity of free water in corn ears in a grain silo cannot be measured directly. The strategy of this study was to calculate the average water change throughout the storage silo by detecting the initial water at the time the grain enters the silo, combined with real-time monitoring of the overall weight drop of the silo. The water potential was calculated by measuring the moisture distribution of each layer at the exit of the silo, and the water potential was positively correlated with the free water activity. If the absolute water potential of the corn was greater than the absolute water potential of the ambient air, the grain moisture migrated to the air and the grain was in a state of desorption. If the situation was reversed, the moisture in the air migrated into the grain kernels to produce adsorption.

**Figure 5:**
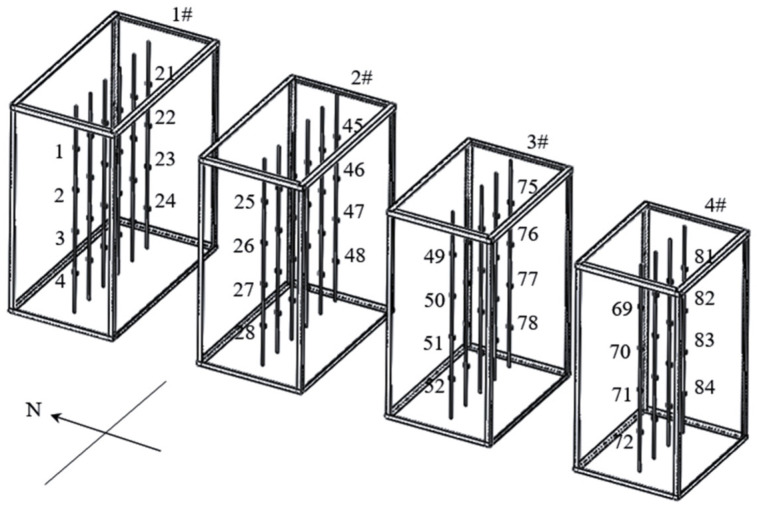
Sensor distribution in grain storage silos (84 temperature sensors labeled 1–84 in four silos).

At the end of the test, the gradient of water potential in each silo was obvious. Moisture was affected by wind direction, with better ventilation on both sides and a faster rate of moisture decline [41]. Table 17, Table 18, Table 19 and Table 20 show the accumulated temperature values collected by 84 temperature sensors in the four grain storage silos. These temperature sensors were distributed in different locations of the grain silos (as shown in Figure 5). By examining the accumulated temperatures, it can be broadly observed that the accumulated temperatures are relatively high in the center of the silos. The corn in the middle had a higher moisture content, which, combined with the fact that the temperature in the middle was also high, made it easy to lead to the production of fungal toxins. It could be seen that the width of the grain silo (grain layer thickness) had an impact on the water potential distribution of corn under natural ventilation conditions, and an unreasonable silo structure could lead to high moisture concentration zones. The high moisture and high heat zone could be predicted by monitoring the absolute water potential in the silo during the grain storage period to ensure the safety of grain storage.

The main contributing factors to the increase in corn mycotoxins in farmers’ grain storage silos include temperature, wind speed, corn ear precipitation rate, and dryness. If the temperature and humidity of agro products are too high, an increase in toxins is likely to occur. Under natural ventilation, the precipitation rate of the corn ear is mainly influenced by temperature and wind speed. During the grain storage process, the temperature changes of the grain piles in the four silos were not very different. During the natural ventilation process, the trend of the precipitation rate curve of grain was increasing with the ambient wind speed, temperature, and absolute water potential curve of grain [41]. Among them, the wind speed had the greatest effect on the precipitation rate of the grain piles. The trends of ambient wind speed curve and precipitation rate curve were generally consistent, both showing low values in January and February. Since the interior of the silo was a large hysteresis system, the overall precipitation rate curve of the grain inside the silo lagged behind the ambient wind speed curve. The absolute water potential of both grain and air increased as the temperature inside the silo gradually increased from −15 °C to 5 °C. The trend of the absolute water potential of corn was the same as that of the ambient temperature, indicating that temperature had a large influence on the absolute water potential. The difference in width caused a large difference in the precipitation rate curves of the four silos. In general, the smaller the width, the greater the precipitation rate. This is more conducive to the drying of the corn ear, thus inhibiting the increase in mycotoxins. Regarding the mycotoxin contamination of maize in Jilin Province that year, aflatoxin B1 was not detected; the highest value of deoxynivalenol was 966.9 μg/kg, with a mean value of 108.2 μg/kg; and the highest value of zearalenone was 42.2 μg/kg, with a mean value of 0.004 μg/kg (according to the Jilin Harvest Grain Quality Survey report). The toxin levels in this experiment were all less than the highest values reported. Mycotoxins in grain silos showed the characteristics of “inverted U-shaped” distribution, indicating that aflatoxins and zearalenone had a tendency to increase in storage and were sensitive to the width of grain silos. For example, regarding the concentration of ZEN in the distribution area of silo 1, the toxin level was close to the maximum allowable value, indicating that there was still a risk of grain storage in farmers’ grain storage silos. Grain storage monitoring and control should be strengthened, and the width of the grain silo design should not be too large (preferably not more than 1.5 m in this study).

## 4. Conclusions

When using rectangular steel mesh ventilated grain storage silos for corn ear storage, the moisture content of corn ears could be reduced to a safe moisture level after 4 months of natural ventilation. This shows that farmers’ scientific grain storage silos can ensure the safety requirements of stored grain mycotoxins under reasonable structure and normal year conditions. The absolute water potential of corn in all four silos at the beginning of the experiment was greater than the absolute water potential of air. This prompted the migration of moisture from the interior of the grain to the air, and the moisture of the corn in the silos gradually decreased to about 14%. The ambient temperature rises so that the absolute water potential of both air and corn gradually increases, but the difference between the two gradually decreases, and finally reaches an equal. Water molecules do not have enough energy to diffuse from the surface into the surrounding air. Moisture is affected by the wind direction, so with better ventilation on both sides, the moisture falls faster. Therefore, the width of the grain silo affects the distribution of water potential and the drying effect of corn under natural ventilation conditions, which in turn affects the production of mycotoxins. For the detection of three mycotoxins in the grain silo, it was shown that the production of mycotoxins was related to the structure of the silos. When the width is too large, there are areas of concentrated infection of mycotoxins such as AFT, DON, and ZEN. Therefore, a reasonable range of silo dimensions should be fully considered in the design of the parameters of grain storage silos. In the middle of the grain silo, there is a potential risk area. It is appropriate to take mechanical ventilation, mechanical turning, and other measures to destroy the opportunity and degree of toxin production in advance. For the sake of food security, scientific grain storage technology and techniques for farmers should be vigorously developed, and this policy fosters the application of scientific grain storage technology for farmers. The above conclusions apply only to the mid-temperate zone at 40–55 degrees north latitude.

## 5. Materials and Methods

### 5.1. Materials

The maize variety was Centaur 335, the largest planted in Jilin Province, with an initial moisture of 26%. The collected maize cobs were mixed samples from the edge and middle of the farm field. They were peeled and left naturally for 48 h to remove ears with lesions, mold, and damage. To ensure that all corn ears in the storage silos had the same level of mycotoxins, the ears were well mixed before entering the silos. Three points in the mixed grain pile were selected for sampling. The content of the three mycotoxins (Aflatoxin, Deoxynivalenol, and Zearalenone) and the rate of mildew were measured separately, and the average value was taken as the content of maize mycotoxins before storage. All samples were stored in silos.

Main reagents and consumables: methanol (analytical purity), acetonitrile (analytical purity), sodium chloride (chemical purity), polyethylene glycol 8000 (analytical purity), glass fiber filter paper, aflatoxin immunoaffinity column (Clover Technology Group Inc., Beijing, China), deoxynivalenol immunoaffinity column (Clover Technology Group Inc., Beijing, China), zearalenone immunoaffinity column (Clover Technology Group Inc., Beijing, China), aflatoxin mixed standard (Sigma-Aldrich China Ltd., Shanghai, China), deoxynivalenol standard (Sigma-Aldrich China Ltd., Shanghai, China), zearalenone standard (Sigma-Aldrich China Ltd., Shanghai, China), phosphate buffer for column crossing, 1.5 mL liquid phase vial set (Agilent), 0.45 um organic phase filtration membrane, 1 mL disposable syringe.

Main instruments and equipment: High performance liquid chromatograph Agilent 1260 with fluorescence and UV detector, pulverizer, high-speed homogenizer, nitrogen blowing apparatus, air pressure pump and pump flow rack, analytical balance (sensitivity 0.001 g), 20 mL glass syringe, 1 mm pore size test sieve.

### 5.2. Homemade Test Silos and Testing Systems

**Figure 6:**
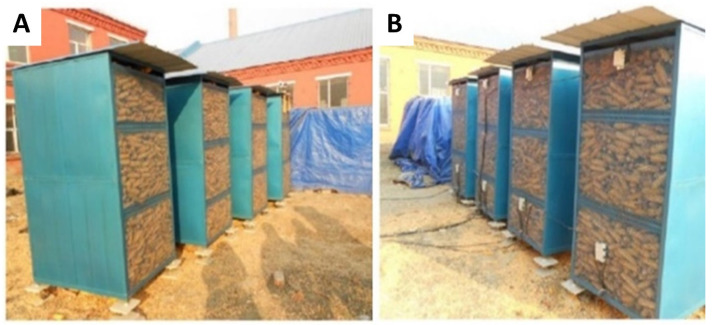
Natural ventilation corn ear storage silos. (**A**) Rectungular steel mesh facing east; (**B**) Rectungular steel mesh facing west.

Four homemade naturally ventilated rectangular corn grain storage silos were used for the experiment (Figure 6). The distribution of many sensors inside them is shown in Figure 5. For the four rectangular steel mesh ventilation silos with different widths, length × width × height were 1 m × 1.8 m × 2 m, 1 m × 1.6 m × 2 m, 1 m × 1.4 m × 2 m, and 1 m × 1.2 m × 2 m, respectively. The tare weights of silos No. 1 to No. 4 were 115.44 kg, 106.08 kg, 96.72 kg, and 87.36 kg, respectively, loaded with 1.85 t, 1.56 t, 1.40 t, and 1.26 t of corn cobs. To test the grain storage effect of various silo sizes in actual operation, the shape of the silo with left and right closed impermeable panels and front and rear permeable mesh panels was used, making the width of the ventilated silo the main factor affecting the grain storage quality (width for left and right closed impermeable panels and length for those with mesh panels).

The testing system mainly included: weighing sensor and meter (Model TQ-ST02, Beijing Shitong Sci-Tech Co., Ltd., Beijing, China), grain moisture measuring instrument (Model PM-8188, KETT, Japan), temperature patrol meter (TR-4, homemade), temperature sensor (DS18B20).

### 5.3. Test Method

The test silos were placed outdoors in a north–south direction. Load cells were added to the foot of the silo to record the overall weight of the silo in real time. The moisture content of the corn in the silo was calculated indirectly through the change in the silo weight. There were 84 temperature sensors distributed in the four grain storage silos, and the numbers were: 24 in silo 1 (the widest), 24 in silo 2, 20 in silo 3, and 16 in silo 4 (the narrowest). The order of arrangement and location was from top to bottom, from west to east. The test started on December 16 of the previous year and ended on April 15 of the following year, with a storage period of about 4 months. During the test, the system automatically collected and stored the data of temperature, weight, and climatic conditions throughout the process. Table 3 shows the average climatic conditions at each stage of the test process. When leaving the silo, samples were taken at each testing point according to the placement of the silo and the height of the grain layer. During the sampling process, the original weight of the silo and the thickness of the grain layer were recorded first, and the corn was sorted out layer by layer starting from the top to the bottom until it reached the temperature sensor position of each layer and then sampled. After reaching the temperature sensor position, the mass of the storage silo (including the remaining corn) and the corresponding thickness of the grain layer were weighed again. When sampling, 8–10 ears of corn were taken as samples near each temperature sensor in the order from west to east. After threshing, samples were put it into the sample bags prepared in advance. The samples were divided into two bags at each sampling point, with each bag containing not less than 500 g. During the sampling process, the remaining corn samples should be kept at the same level as the temperature sensor.

### 5.4. Calculation of the Absolute Water Potential of the Grain Pile

The absolute water potentials of air and grain were used to calculate the absolute water potential of corn during natural ventilation and the absolute water potential of air under experimental conditions, respectively, to assess the absolute water potential variation and distribution of grain piles and the drying effect of grain silos.

(1) $$E_{ja}=8.31\times (t_{a}+273)\times \ln(100\times \exp(\frac{87.72\times \mathrm{lg}(RH_{a})+0.9845\times (1737.1-\frac{474242}{273+t_{g}})-270.57}{87.72})\times 133.3)/18$$

(2) $$E_{jg}=8.31\times (t_{g}+273)\times \ln(\exp(\frac{(\frac{D_{0}}{222}\times (e^{\frac{B_{1}-M}{A_{1}}}-e^{\frac{B_{2}-M}{A_{2}}})+0.9845)\times (1737.1-\frac{474242}{273+t_{g}})+D_{0}\times (1-e^{\frac{B_{1}-M}{A_{1}}})-68.57}{87.72})\times 133.3)/18$$

where *E_ja_* was the absolute water potential of air, kJ/kg; *E_jg_* was the absolute water potential of grain, kJ/kg; *M* was the wet basis moisture content of grain, %; *t_g_* was the temperature of grain, °C; *RH_a_* was the relative humidity of air, %; *t_a_* was the temperature of air, °C; and *A*_1_, *A*_2_, *B*_1_, *B*_2_, and *D*_0_ were the desorption parameters of corn, 4.393, 4.845, 7.843, 3.858, and 203.892, respectively.

### 5.5. Determination of Aflatoxin Content

The aflatoxin content in food was determined regarding the Chinese national standard GB 5009.22–2016. The sample was extracted with methanol–water, the extract was filtered and diluted, and the filtrate was purified by immunoaffinity chromatography containing aflatoxin-specific antibodies. This antibody was specific for aflatoxin B1, B2, G1, and G2. Aflatoxin was cross-linked to the antibody in the chromatography medium. Impurities were removed from the immunoaffinity column with water or Tween-20/phosphate-buffered solution (PBS). Elution was performed with methanol through the immunoaffinity chromatography column. The eluate was passed through the column of HPLC. It was then derivatized using an AURA photochemical derivatization cell and detected by a fluorescence detector and quantified by external standard method.

### 5.6. Determination of Deoxynivalenol Content

The determination of deoxynivalenol in food was carried out regarding the Chinese national standard GB 5009.111–2016. Deoxynivalenol was extracted from the sample. After purification and concentration by immunoaffinity column, the sample was determined by HPLC with UV detector and quantified by external standard method.

### 5.7. Determination of Zearalenone Content

The determination of zearalenone in cereals was carried out regarding the national standard GB 5009.209–2016. Zearalenone was extracted from the sample with acetonitrile–water, and the extract was cleaned up and concentrated by an immunoaffinity column. The determination was performed by HPLC with a fluorescence detector and quantified by external standard method.

The method validation involved in the above six mycotoxin assays is shown in Table 21.

### 5.8. Mold Rate

Chinese national standard GB1353-2009 was used for the mold rate to make clear provisions—raw mold particles need to be ≤2%.

### 5.9. Statistical Analysis

Statistical analysis was performed using Origin 9.0, and data were expressed as mean standard deviation SD (*n* = 3). Significant differences between means (*p* < 0.05) were investigated by Tukey’s test, using one-way ANOVA with SPSS 17.0.

## Figures and Tables

**Table 1:** Average moisture change during grain storage (unit: %).

Time	Silo 1	Silo 2	Silo 3	Silo 4
The second half of December	25.40	24.88	25.27	24.55
The first half of January	24.75	24.49	23.97	23.73
The second half of January	24.10	23.61	23.06	22.66
The first half of February	22.37	21.59	20.95	20.53
The second half of February	21.19	20.24	19.53	18.70
The first half of March	20.66	19.61	18.48	18.01
The second half of March	18.36	16.77	15.53	16.17
The first half of April	15.06	13.09	12.01	13.69

**Table 2:** The absolute water potential energy of corn in each silo during natural ventilation (unit: kJ/kg).

Time	Silo 1	Silo 2	Silo 3	Silo 4
The second half of December	635.37	637.27	652.04	647.72
The first half of January	649.13	652.46	650.66	650.36
The second half of January	653.21	655.01	654.25	652.44
The first half of February	672.39	667.69	656.38	670.88
The second half of February	697.12	698.42	695.99	693.21
The first half of March	746.15	747.81	743.61	744.14
The second half of March	798.69	791.33	798.22	792.23
The first half of April	830.02	814.49	807.11	827.47

**Table 3:** The absolute water potential energy of air (unit: kJ/kg).

Time	Average Temperature (°C)	Average Humidity (%)	Average Wind Speed (m/s)	Average Absolute Potential Energy (kJ/kg)
The second half of December	−11.12	35	0.22	619.75
The first half of January	−11.21	33	0.32	615.21
The second half of January	−10.93	30	0.33	614.54
The first half of February	−9.50	28	0.78	626.48
The second half of February	−7.48	34	0.76	661.43
The first half of March	−3.11	37	0.43	716.78
The second half of March	1.49	46	1.14	783.38
The first half of April	6.38	33	1.19	822.14
Average value	−5.69	34	0.65	682.46

**Table 4:** Results of the determination of aflatoxin content in corn samples from storage silos (unit: μg/kg).

	Sample No.	AFB1	AFB2	Sample No.	AFB1	AFB2	Sample No.	AFB1	AFB2	Sample No.	AFB1	AFB2
Silo 1												
Top	1–1T	0.25 ± 0.01	/		/	/		/	/		/	/
Layer 1	1–1	0.35 ± 0.03	/	1–9	0.40 ± 0.04	0.05 ± 0.00	1–13	0.38 ± 0.02	/	1–21	0.32 ± 0.03	/
Layer 2	1–2	0.68 ± 0.07	0.10 ± 0.01	1–10	0.75 ± 0.02	0.43 ± 0.01	1–14	0.64 ± 0.03	0.45 ± 0.02	1–22	0.59 ± 0.01	/
Layer 3	1–3	0.86 ± 0.02	0.40 ± 0.04	1–11	0.95 ± 0.01	0.75 ± 0.03	1–15	0.90 ± 0.10	0.65 ± 0.05	1–23	0.85 ± 0.09	0.25 ± 0.01
Layer 4	1–4	0.72 ± 0.06	0.25 ± 0.02	1–12	0.78 ± 0.00	0.60 ± 0.04	1–16	0.68 ± 0.02	0.51 ± 0.02	1–24	0.62 ± 0.08	0.10 ± 0.00
Bottom	1–4B	0.48 ± 0.01	/	1–12B	0.62 ± 0.01	/	1–16B	0.58 ± 0.05	/	1–24B	0.45 ± 0.06	/
Silo 2												
Top	2–25T	/	/	/	/	/	/	/	/	/	/	/
Layer 1	2–25	0.30 ± 0.02	/	2–33	0.35 ± 0.01	/	2–37	0.32 ± 0.07	/	2–45	0.28 ± 0.00	/
Layer 2	2–26	0.58 ± 0.03	/	2–34	0.63 ± 0.04	0.05 ± 0.01	2–38	0.57 ± 0.05	0.10 ± 0.02	2–46	0.51 ± 0.08	/
Layer 3	2–27	0.71 ± 0.07	0.11 ± 0.01	2–35	0.82 ± 0.12	0.49 ± 0.10	2–39	0.75 ± 0.10	0.55 ± 0.08	2–47	0.68 ± 0.04	0.05 ± 0.01
Layer 4	2–28	0.62 ± 0.05	/	2–36	0.67 ± 0.10	0.35 ± 0.06	2–40	0.65 ± 0.08	0.46 ± 0.10	2–48	0.60 ± 0.07	/
Bottom	2–28B	0.48 ± 0.03	/	2–36B	0.48 ± 0.06	/	2–40B	0.46 ± 0.06	/	2–48B	0.39 ± 0.01	/
Silo 3												
Top	3–49T	/	/		/	/		/	/		/	/
Layer 1	3–49	/	/	3–57	/	/	3–61	/	/	3–65	/	/
Layer 2	3–50	0.28 ± 0.01	/	3–58	0.55 ± 0.03	0.04 ± 0.00	3–62	0.49 ± 0.03	/	3–66	0.45 ± 0.04	/
Layer 3	3–51	0.40 ± 0.03	0.11 ± 0.01	3–59	0.70 ± 0.06	0.35 ± 0.07	3–63	0.65 ± 0.11	0.25 ± 0.06	3–67	0.60 ± 0.09	0.05 ± 0.01
Layer 4	3–52	0.32 ± 0.01	/	3–60	0.60 ± 0.04	0.10 ± 0.01	3–64	0.58 ± 0.09	0.03 ± 0.01	3–68	0.55 ± 0.04	/
Bottom	3–52B	/	/	3–60B	0.26 ± 0.01	/	3–64B	0.24 ± 0.02	/	3–68B	/	/
Silo 4												
Top	4–69T	/	/		/	/		/	/		/	/
Layer 1	4–69	/	/	4–73	/	/	4–77	/	/	4–81	/	/
Layer 2	4–70	0.20 ± 0.01	/	4–74	0.44 ± 0.03	/	4–78	0.36 ± 0.02	/	4–82	0.28 ± 0.02	/
Layer 3	4–71	0.35 ± 0.03	/	4–75	0.50 ± 0.08	0.17 ± 0.01	4–79	0.45 ± 0.07	/	4–83	0.30 ± 0.02	/
Layer 4	4–72	0.24 ± 0.00	/	4–76	0.34 ± 0.02	0.10 ± 0.00	4–80	0.28 ± 0.01	/	4–84	0.30 ± 0.03	/
Bottom	4–72B	/	/	4–76B	/	/	4–80B	/	/	4–84B	/	/

Note: “/” in the table means not detected. AFG1 and AFG2 were not detected before and after the experiment, so they are not reflected in the table.

**Table 5:** Results of the determination of deoxynivalenol (DON) content in corn samples from grain storage silos (unit: μg/kg).

	Sample No.	DON	Sample No.	DON	Sample No.	DON	Sample No.	DON
Silo 1								
Top	1–1T	707.92 ± 0.81						
Layer 1	1–1	714.20 ± 3.95	1–9	718.96 ± 5.01	1–13	721.46 ± 5.83	1–21	710.68 ± 4.49
Layer 2	1–2	715.46 ± 7.01	1–10	724.50 ± 8.91	1–14	733.62 ± 8.63	1–22	719.80 ± 6.61
Layer 3	1–3	721.31 ± 8.56	1–11	730.81 ± 9.14	1–15	741.51 ± 7.91	1–23	727.65 ± 8.19
Layer 4	1–4	718.78 ± 4.89	1–12	726.43 ± 6.01	1–16	731.63 ± 5.68	1–24	720.77 ± 6.74
Bottom	1–4B	694.34 ± 6.90	1–12B	689.85 ± 6.81	1–16B	701.49 ± 7.77	1–24B	705.38 ± 6.36
Silo 2								
Top	2–25T	689.30 ± 0.61						
Layer 1	2–25	701.68 ± 7.91	2–33	696.53 ± 6.83	2–37	690.64 ± 7.66	2–45	684.11 ± 7.80
Layer 2	2–26	708.76 ± 7.89	2–34	713.54 ± 8.43	2–38	700.35 ± 8.02	2–46	695.40 ± 8.26
Layer 3	2–27	715.41 ± 9.25	2–35	736.26 ± 11.05	2–39	720.50 ± 11.89	2–47	711.47 ± 11.29
Layer 4	2–28	712.53 ± 10.27	2–36	722.87 ± 9.31	2–40	716.96 ± 8.96	2–48	700.84 ± 8.70
Bottom	2–28B	684.26 ± 7.13	2–36B	675.11 ± 8.31	2–40B	670.24 ± 7.73	2–48B	666.21 ± 7.87
Silo 3								
Top	3–49T	645.42 ± 1.71						
Layer 1	3–49	650.33 ± 6.11	3–57	659.22 ± 6.52	3–61	664.16 ± 5.93	3–65	653.20 ± 6.12
Layer 2	3–50	657.15 ± 6.00	3–58	665.31 ± 6.85	3–62	670.20 ± 6.06	3–66	658.67 ± 5.45
Layer 3	3–51	675.30 ± 8.81	3–59	685.45 ± 9.06	3–63	689.75 ± 7.88	3–67	670.76 ± 9.37
Layer 4	3–52	666.47 ± 8.39	3–60	672.91 ± 8.15	3–64	680.03 ± 9.05	3–68	660.36 ± 8.29
Bottom	3–52B	620.94 ± 5.96	3–60B	629.47 ± 6.69	3–64B	630.15 ± 7.75	3–68B	616.23 ± 6.60
Silo 4								
Top	4–69T	620.44 ± 0.77						
Layer 1	4–69	653.02 ± 9.97	4–73	661.33 ± 12.16	4–77	641.42 ± 9.33	4–81	638.37 ± 10.54
Layer 2	4–70	655.41 ± 12.03	4–74	669.23 ± 11.85	4–78	649.75 ± 12.61	4–82	640.14 ± 12.19
Layer 3	4–71	660.44 ± 13.17	4–75	680.35 ± 11.99	4–79	670.99 ± 13.93	4–83	650.15 ± 13.15
Layer 4	4–72	658.86 ± 12.77	4–76	674.36 ± 13.72	4–80	650.65 ± 13.28	4–84	642.82 ± 14.11
Bottom	4–72B	618.29 ± 7.81	4–76B	611.82 ± 6.58	4–80B	607.10 ± 7.99	4–84B	600.33 ± 7.62

**Table 6:** Results of the determination of zearalenone (ZEN) content in corn samples from grain storage silos (unit: μg/kg).

	Sample No.	ZEN	Sample No.	ZEN	Sample No.	ZEN	Sample No.	ZEN
Silo 1								
Top	1–1T	36.61 ± 1.24						
Layer 1	1–1	37.32 ± 0.08	1–9	37.35 ± 0.26	1–13	36.93 ± 0.22	1–21	37.22 ± 0.20
Layer 2	1–2	39.34 ± 2.13	1–10	43.45 ± 1.99	1–14	42.45 ± 1.37	1–22	41.21 ± 1.75
Layer 3	1–3	40.12 ± 3.01	1–11	47.09 ± 4.25	1–15	46.11 ± 3.93	1–23	40.25 ± 3.65
Layer 4	1–4	38.28 ± 0.41	1–12	39.00 ± 0.72	1–16	38.75 ± 0.43	1–24	37.78 ± 0.72
Bottom	1–4B	36.30 ± 0.66	1–12B	37.46 ± 1.07	1–16B	36.67 ± 0.56	1–24B	35.48 ± 0.91
Silo 2								
Top	2–25T	34.41 ± 2.03						
Layer 1	2–25	34.32 ± 0.63	2–33	35.21 ± 0.46	2–37	34.89 ± 0.49	2–45	34.08 ± 0.46
Layer 2	2–26	35.27 ± 0.74	2–34	36.38 ± 0.57	2–38	35.88 ± 0.65	2–46	34.98 ± 0.56
Layer 3	2–27	37.94 ± 3.06	2–35	42.11 ± 2.69	2–39	38.67 ± 2.08	2–47	36.45 ± 1.69
Layer 4	2–28	35.40 ± 1.81	2–36	38.24 ± 0.94	2–40	36.42 ± 1.26	2–48	35.11 ± 1.59
Bottom	2–28B	33.09 ± 0.01	2–36B	33.04 ± 0.02	2–40B	32.67 ± 0.01	2–48B	32.23 ± 0.03
Silo 3								
Top	3–49T	33.34 ± 0.98						
Layer 1	3–49	34.67 ± 0.44	3–57	34.22 ± 0.31	3–61	34.55 ± 0.39	3–65	33.80 ± 0.42
Layer 2	3–50	35.10 ± 1.03	3–58	36.67 ± 0.88	3–62	36.17 ± 0.93	3–66	34.63 ± 1.04
Layer 3	3–51	35.15 ± 2.77	3–59	40.03 ± 3.07	3–63	38.14 ± 1.53	3–67	35.34 ± 2.07
Layer 4	3–52	34.86 ± 2.37	3–60	38.81 ± 2.49	3–64	36.70 ± 1.83	3–68	34.22 ± 1.63
Bottom	3–52B	33.10 ± 0.41	3–60B	33.45 ± 0.19	3–64B	33.44 ± 0.32	3–68B	32.91 ± 0.20
Silo 4								
Top	4–69T	31.56 ± 1.01						
Layer 1	4–69	31.06 ± 0.51	4–73	31.78 ± 0.39	4–77	32.01 ± 0.49	4–81	31.21 ± 0.53
Layer 2	4–70	31.91 ± 1.01	4–74	33.85 ± 2.03	4–78	32.93 ± 0.68	4–82	31.50 ± 0.40
Layer 3	4–71	32.72 ± 1.89	4–75	35.81 ± 2.07	4–79	36.05 ± 1.33	4–83	32.22 ± 2.71
Layer 4	4–72	32.15 ± 1.65	4–76	34.62 ± 1.09	4–80	34.38 ± 2.05	4–84	31.83 ± 1.13
Bottom	4–72B	30.45 ± 0.72	4–76B	31.74 ± 0.57	4–80B	30.78 ± 0.62	4–84B	30.09 ± 0.81

**Table 7:** Average aflatoxin levels in each layer of grain storage silos (unit: μg/kg).

	Silo 1	Silo 2	Silo 3	Silo 4
Top	0.06 ± 0.01	0	0	0
Layer 1	0.38 ± 0.03	0.31 ± 0.01	0	0
Layer 2	0.88 ± 0.07	0.61 ± 0.05	0.45 ± 0.03	0.32 ± 0.02
Layer 3	1.40 ± 0.09	1.04 ± 0.11	0.78 ± 0.11	0.44 ± 0.05
Layer 4	1.07 ± 0.07	0.85 ± 0.09	0.55 ± 0.05	0.32 ± 0.02
Bottom	0.53 ± 0.03	0.45 ± 0.03	0.13 ± 0.01	0

**Table 8:** Levels of aflatoxin in each column of the grain storage silos (unit: μg/kg).

	Silo 1	Silo 2	Silo 3	Silo 4
West 1 column	0.45 ± 0.03	0.31 ± 0.02	0.12 ± 0.01	0.09 ± 0.01
West 2 column	0.59 ± 0.02	0.43 ± 0.06	0.29 ± 0.03	0.17 ± 0.02
West 3 column	0.60 ± 0.04	0.48 ± 0.07	0.28 ± 0.04	0.14 ± 0.01
West 4 column	0.45 ± 0.04	0.36 ± 0.03	0.24 ± 0.03	0.13 ± 0.01

**Table 9:** The average contents of aflatoxin in each storage silo and the ratio of difference with the content in silo 1.

Indicators	Silo 1	Silo 2	Silo 3	Silo 4
Toxin content (μg/kg)	0.36 ± 0.02a	0.27 ± 0.03b	0.16 ± 0.02c	0.09 ± 0.01d
Ratio of difference (%)	0	25.14	56.08	75.14

Note: The differences between the values with different letters in the same row are significant (*p* < 0.05).

**Table 10:** Average deoxynivalenol levels in each layer of grain storage silos (unit: μg/kg).

	Silo 1	Silo 2	Silo 3	Silo 4
Top	707.92 ± 0.81	689.30 ± 0.61	645.42 ± 1.71	620.44 ± 0.77
Layer 1	716.33 ± 4.82	693.24 ± 7.55	656.73 ± 6.17	648.54 ± 10.50
Layer 2	723.35 ± 7.79	704.51 ± 8.15	662.83 ± 6.09	653.63 ± 12.17
Layer 3	730.32 ± 8.45	720.91 ± 10.87	680.32 ± 8.78	665.48 ± 13.06
Layer 4	724.40 ± 5.83	713.30 ± 9.31	669.94 ± 8.47	656.67 ± 13.47
Bottom	697.77 ± 6.96	673.96 ± 7.76	624.20 ± 6.75	609.39 ± 7.50

**Table 11:** Contents of deoxynivalenol in each column of the grain storage silos (unit: μg/kg).

	Silo 1	Silo 2	Silo 3	Silo 4
West 1 column	712.00 ± 5.35	700.82 ± 7.18	652.60 ± 6.16	644.41 ± 9.51
West 2 column	718.11 ± 7.18	708.86 ± 8.79	663.98 ± 5.96	659.42 ± 11.26
West 3 column	725.94 ± 7.16	699.74 ± 8.85	667.36 ± 6.84	643.98 ± 11.43
West 4 column	716.86 ± 6.48	691.61 ± 8.79	651.84 ± 7.16	634.46 ± 11.42

**Table 12:** The average contents of deoxynivalenol in each storage silo and the ratio of difference with the content in silo 1.

Indicators	Silo 1	Silo 2	Silo 3	Silo 4
Toxin content (μg/kg)	717.91 ± 12.95a	700.63 ± 18.31a	658.10 ± 20.25b	645.42 ± 22.68b
Ratio of difference (%)	0	2.12	8.23	10.05

Note: The differences between the values with different letters in the same row are significant (*p* < 0.05).

**Table 13:** Average zearalenone contents in each layer of grain storage silos (unit: μg/kg).

	Silo 1	Silo 2	Silo 3	Silo 4
Top	36.61 ± 1.24	34.41 ± 2.03	33.34 ± 0.98	31.56 ± 1.01
Layer 1	37.21 ± 0.19	34.63 ± 0.51	34.31 ± 0.39	31.52 ± 0.48
Layer 2	41.61 ± 1.81	35.63 ± 0.63	35.64 ± 0.97	32.55 ± 1.03
Layer 3	43.39 ± 3.71	38.79 ± 2.38	37.17 ± 2.36	34.20 ± 2.00
Layer 4	38.45 ± 0.57	36.29 ± 1.40	36.15 ± 2.08	33.25 ± 1.48
Bottom	36.48 ± 0.80	32.76 ± 0.36	33.23 ± 0.28	30.77 ± 0.68

**Table 14:** Contents of zearalenone in each column of the grain storage silos (unit: μg/kg).

	Silo 1	Silo 2	Silo 3	Silo 4
West 1 column	38.00 ± 1.25	35.07 ± 1.38	34.37 ± 1.33	31.64 ± 1.13
West 2 column	40.87 ± 1.66	37.00 ± 0.94	36.64 ± 1.39	33.56 ± 1.23
West 3 column	40.18 ± 1.30	35.71 ± 0.90	35.80 ± 1.00	33.23 ± 1.03
West 4 column	38.39 ± 1.45	34.60 ± 0.90	34.18 ± 1.07	31.37 ± 1.12

**Table 15:** The average contents of zearalenone in each storage silo and the ratio of difference with the content in silo 1.

Indicators	Silo 1	Silo 2	Silo 3	Silo 4
Toxin content (μg/kg)	38.96 ± 3.18a	35.42 ± 2.29ab	34.97 ± 1.93ab	32.31 ± 1.64b
Ratio of difference (%)	0	9.62	10.48	17.68

Note: The differences between the values with different letters in the same row are significant (*p* < 0.05).

**Table 16:** The rate of corn mold in the silo at the time of leaving the warehouse (unit: %).

	Silo 1	Silo 2	Silo 3	Silo 4
Maximum value	0.387	0.547	0.143	0.248
Minimum value	0	0.009	0	0
Difference	0.387	0.539	0.143	0.248
Average value	0.088	0.125	0.053	0.062

**Table 17:** Values of the accumulated temperature obtained by temperature sensors at different points in grain storage silo 1 (unit: °C).

Sample No.	Accumulated Temperature	Sample No.	Accumulated Temperature	Sample No.	Accumulated Temperature	Sample No.	Accumulated Temperature	Sample No.	Accumulated Temperature	Sample No.	Accumulated Temperature
1	−2013.1	5	−2012.4	9	−2000.3	13	−2064.0	17	−2164.6	21	−2142.7
2	−2137.6	6	−2120.6	10	−2084.8	14	−2104.3	18	−2253.9	22	−2279.4
3	−2271.6	7	−2217.7	11	−2181.7	15	−2166.0	19	−2157.4	23	−2203.9
4	−2328.7	8	−2310.5	12	−2225.6	16	−2258.9	20	−2283.6	24	−2268.7

**Table 18:** Values of the accumulated temperature obtained by temperature sensors at different points in grain storage silo 2 (unit: °C).

Sample No.	Accumulated Temperature	Sample No.	Accumulated Temperature	Sample No.	Accumulated Temperature	Sample No.	Accumulated Temperature	Sample No.	Accumulated Temperature	Sample No.	Accumulated Temperature
25	−2026.5	29	−2035.6	33	−2028.0	37	−2034.9	41	−2084.3	45	−2149.3
26	−2127.9	30	−2119.7	34	−2117.9	38	−2073.3	42	−2115.1	46	−2106.7
27	−2216.3	31	−2194.1	35	−2137.6	39	−2166.4	43	−2112.7	47	−2180.3
28	−2252.9	32	−2232.4	36	−2244.1	40	−2209.1	44	−2179.2	48	−2197.7

**Table 19:** Values of the accumulated temperature obtained by temperature sensors at different points in grain storage silo 3 (unit: °C).

Sample No.	Accumulated Temperature	Sample No.	Accumulated Temperature	Sample No.	Accumulated Temperature	Sample No.	Accumulated Temperature	Sample No.	Accumulated Temperature
49	−2139.4	53	−2018.4	57	−1981.6	61	−2019.6	65	−2052.8
50	−2139.1	54	−2111.2	58	−2110.1	62	−2091.2	66	−2061.8
51	−2169.0	55	−2174.4	59	−2152.3	63	−2134.7	67	−2217.1
52	−2263.8	56	−2244.4	60	−2173.0	64	−2160.6	68	−2043.5

**Table 20:** Values of the accumulated temperature obtained by temperature sensors at different points in grain storage silo 4 (unit: °C).

Sample No.	Accumulated Temperature	Sample No.	Accumulated temperature	Sample No.	Accumulated Temperature	Sample No.	Accumulated Temperature
69	−2045.7	73	−2045.2	77	−2036.2	81	−2065.8
70	−2185.1	74	−2159.9	78	−2146.4	82	−2045.1
71	−2207.1	75	−2178.1	79	−2134.7	83	−2096.7
72	−2249.6	76	−2205.5	80	−2201.1	84	−2135.5

**Table 21:** Summary of the method validation.

Mycotoxin ^a^	AFB1	AFB2	AFG1	AFG2	ZEN	DON
Coefficient of correlation (R^2^)	0.999	0.999	1.000	0.999	0.999	0.999
Range (ng/mL)	0.1–40	0.03–12	0.1–40	0.03–12	10–500	100–5000
Spiked level (μg/kg)	10	10	10	10	30.0	300.0
Recovery (%)	93.7	105.3	96.9	101.1	97.5	96.6
RSD (%) *	10.2	17.8	11.4	16.1	2.7	2.2
LOD (μg/kg)	0.03	0.01	0.03	0.01	5	100
LOQ (μg/kg)	0.1	0.03	0.1	0.03	17	200

Note: ^a^ AFB1, AFB2, AFG1, AFG2: aflatoxins B1, B2, G1, and G2; ZEN: zearalenone; DON: deoxynivalenol. RSD: relative standard deviation; LOD: limit of detection; LOQ: limit of quantification. * This value was calculated from 3 replicates of mycotoxin analysis.

## Data Availability

Data is contained within this article.
